# Optically-assisted thermophoretic reversible assembly of colloidal particles and *E. coli* using graphene oxide microstructures

**DOI:** 10.1038/s41598-022-07588-4

**Published:** 2022-03-07

**Authors:** Jostine Puthenveetil Joby, Suman Das, Praveenkumar Pinapati, Benoît Rogez, Guillaume Baffou, Dhermendra K. Tiwari, Sudhir Cherukulappurath

**Affiliations:** 1 School of Physical and Applied Sciences, Goa University, Taleigao Plateau, Goa 403206 India; 2 Department of Biotechnology, Goa University, Taleigao Plateau, Goa 403206 India; 3 Institut Fresnel, CNRS, Aix Marseille University, Centrale Marseille, Marseille, France

**Keywords:** Biological techniques, Biophysics, Materials science, Nanoscience and technology, Optics and photonics, Physics

## Abstract

Optically-assisted large-scale assembly of nanoparticles have been of recent interest owing to their potential in applications to assemble and manipulate colloidal particles and biological entities. In the recent years, plasmonic heating has been the most popular mechanism to achieve temperature hotspots needed for extended assembly and aggregation. In this work, we present an alternative route to achieving strong thermal gradients that can lead to non-equilibrium transport and assembly of matter. We utilize the excellent photothermal properties of graphene oxide to form a large-scale assembly of silica beads. The formation of the assembly using this scheme is rapid and reversible. Our experiments show that it is possible to aggregate silica beads (average size 385 nm) by illuminating thin graphene oxide microplatelet by a 785 nm laser at low intensities of the order of 50–100 µW/µm^2^. We further extend the study to trapping and photoablation of *E. coli* bacteria using graphene oxide. We attribute this aggregation process to optically driven thermophoretic forces. This scheme of large-scale assembly is promising for the study of assembly of matter under non-equilibrium processes, rapid concentration tool for spectroscopic studies such as surface-enhanced Raman scattering and for biological applications.

## Introduction

Nonequilibrium thermodynamic transport processes have been of significant interest in the recent years owing to their applications involving thermodiffusive flows^1^, large scale assembly^2,3^, and segregation of DNA^4^, micro or nanoparticles^5,6^. Such transport and assembly of particles usually harness strong gradients of either temperature, molar concentrations, electric field or optical fields. For example, dielectrophoresis and electrophoresis involves particle mass transport and segregation using AC or DC electric fields. Dielectrophoresis, in particular, involves strong field induced charges due to non-uniform electric fields that drives the particles towards the highest field gradient^7^. This technique has been used for biological applications such as cell trapping and sorting^8^, manipulation of proteins and viruses^9,10^ as well as for SERS based sensing techniques^11–13^. However, electrophoretic and dielectrophoretic methods have certain limitations such as joule heating of electrodes, strong dependence on the electrical conductivity of the buffer solution, frequent damage of samples and material properties of particles. Optical tweezers have been successfully used not only to trap and manipulate subwavelength particles at specific location but also for large-scale assembly on substrates^14–17^. Conventional optical tweezers involve a tightly focused light beam that generates a strong gradient force capable of holding microparticles. However, for stable subwavelength particle trapping, the intensity of the electric field has to be much larger than other competitive forces such as scattering and convective forces, which can in turn damage the sample that is being trapped. In this regard, surface plasmon-based trapping schemes have been demonstrated to be more efficient owing to the strong near-field that is generated around plasmonic nanoparticles upon illumination^18–21^. There have been several reports of plasmonics-based optical trapping of particles of different sizes varying from microbeads to quantum dots^22,23^. However, due to the short range of plasmonic near-field, large-scale assembly of colloidal particles using plasmonic forces requires either arrays of nanostructures^24,25^ or continuous thin metal films where propagating surface plasmons (surface plasmon polaritons) can be excited^26^. Moreover, the geometrical size of the optical beam used for trapping puts a limit on the trapping area. Self-assembly of mesoscopic particles using microbubbles generated by optical beams have been demonstrated^27,28^. On the other hand, temperature gradients have the capability to generate long-range effects that can be harnessed to create mass transport and aggregation. Thermal effects such as convection, thermophoresis, Marangoni and thermoelectric forces have been used as an effective tool for local control of particle flow and assembly^3,5,29–34^. In particular, optically-generated thermal effects can be beneficial in providing a controlled thermal gradient which in turn can drive the colloidal particles to the required site. For example, Garcés-Chávez et al. have reported the use of optical and plasmonic forces to create thermal gradients that lead to large scale assembly of colloidal particles^26^. The authors showed that extended assembly of colloidal particles by optically-induced thermal gradients is an interplay between the convective forces and thermophoresis. Convection, in general, results in the transfer of hotter fluids to colder regions thereby carrying the colloidal particles along with them circulation of fluids due to temperature gradients. Depending on the strength of the temperature gradient and size of the fluidic chamber, convective forces can either dominate the flow or can be insignificant. Thermophoresis, on the other hand, is a non-equilibrium thermal diffusion process (Soret effect) where the colloids are driven to either hot or cold regions of the fluid^29,35,36^. The direction of thermophoretic flow can depend on the temperature, on the fluid properties (composition, pH, ionicity), the nanoparticle size and the nature of the particle. Under thermophoretic process, the current density of flow is given by the relation^29^:

1 $$J = - D\nabla c - cD_{T} \nabla T$$

The first term is related to the standard diffusion which is dependent on the concentration gradient while the second term is proportional to the temperature gradient. Here, $D$ is the diffusion coefficient (also called Brownian diffusion coefficient) and $D_{T}$ is the thermal diffusion coefficient (although it does not have dimensions of diffusion coefficient). $D_{T}$ is in fact the drift mobility due to the thermal gradient and hence the drift velocity is given by $v_{T} = - D_{T} \nabla T$. Due to this drift, there is a concentration change in the locality of the thermal gradient and in the steady state:

2 $$\frac{\nabla c}{c} = - S_{T} \nabla T$$

where $S_{T} = \frac{{D_{T} }}{D}$ is called the Soret coefficient. For $S_{T} > 0$, the particles move from the hot region to the cold region while for $S_{T} < 0$, the reverse flow is observed. The value of $S_{T}$ is very system-specific and can be manipulated by changing the properties of the particle or the solvent. For instance, a reversal in the sign of Soret coefficient of colloidal polystyrene beads in a polymeric (PEG) solution simply by altering the concentration of the polymer was reported earlier^33,37^. Thus, it is possible to aggregate the suspended colloids or to release them simply by altering the properties of the solvent. Plasmonic heating by illumination of noble metals can be harnessed to induce a temperature gradient for trapping and colloidal assembly applications. A low power laser based plasmon-enhanced thermophoretic technique to generate reversible assembly of plasmonic nanoparticles on gold was reported^38^. More recently, it was shown that large-scale assembly of silica beads was possible by optically assisted heating of gold microplates^39^. By illuminating the gold microplatelet using evanescent waves, joule heating of the gold structure caused a thermal gradient that generated the assembly of the micron sized beads into hexagonal packing structures. Optically-assisted thermoelectric tweezers were reported by Linhan Li and coworkers wherein electric charges were utilized in addition to optically driven thermophoretic forces for the assembly of nanoparticles^40^. Efficient trapping of nanoparticles, quantum dots and cell specimens was demonstrated using this novel technique^31,33,41,42^. All these schemes however depend on the thermal response of plasmonic metals such as gold on under illumination with laser light. The fabrication of plasmonic nanostructures and films require expensive, time consuming and sometimes sophisticated instruments such as e-beam lithography or focused-ion beam milling. Furthermore, metallic structures tend to be less transmissive thereby making it necessary to work in reflection mode for visualizing the trapping events. We have recently shown that it is possible to trap nanoparticles and quantum dots using graphene oxide (GO) microplatelets at very low intensities^43^. The unique optical properties of GO and inexpensive synthesis methods makes it a promising material for such applications^44–48^. The excellent photothermal properties of carbon-based nanomaterials can be harnessed to generate strong thermal gradients that can lead to convection and thermophoresis. Two-dimensional (2D) carbon nanostructures such as graphene and carbon nanotubes have been demonstrated to be suitable materials for biological applications such as photothermal therapy and bacterial destruction^49–51^. Furthermore, there have been several reports of novel SERS schemes using graphene oxide and its composites^52,53^.

Graphene and its derivatives have a broad absorption band in the visible to near-infrared making them efficient thermal emitters. Microparticle aggregation and manipulation using single walled carbon nanotubes with relatively low optical intensities was demonstrated by Qian and co-workers^54^. However, there have been no reports of using 2D materials such as graphene or GO as thermal sources for large-scale optical assembly of colloidal particles.

In this work, we make use of the photothermal properties of GO microplatelets to generate large scale reversible assembly of colloidal nano and microparticles. GO, synthesized chemically using a modified Hummers method, is drop-casted onto a glass slide. The colloidal solution of silica beads is then dispersed on the substrate and illuminated with a focused laser light of 785 nm wavelength. It was observed that at specific powers, the colloidal particles assemble on the GO sheet in a specific pattern. We quantified the rate of growth of the assembly with power as well as with time at constant powers. The assembly process is rapid and the particles can be released back into the solution by switching the laser off. In order to extend the capability of the scheme, we used the thermal gradient generated by GO illuminated by laser to trap and aggregate *E. coli* bacteria on the GO leading to their photothermal destruction. Our studies show that the bacteria are photo-ablated upon illumination of the laser in the presence of GO.

## Experimental section

### Preparation of GO

GO was prepared by oxidation of graphite flakes using improved Hummer`s method^55^. A concentrated mixture of 225 ml H_2_SO_4_ and 25 ml H_3_PO_4_ (9:1 vol. equivalent) was added to another mixture of 18 g KMnO_4_ and 3 g graphite flakes (6:1 wt. equivalent) in an ice bath since the reaction is exothermic in nature. The mixture was heated at 55 °C for 12 h with constant stirring. After cooling to room temperature, 200 ml of ice cold 30% H_2_O_2_ was added to the reflexed solution in an ice bath under constant stirring and the solution was left for separation overnight. Upper layer of the solution was decanted and lower layer was washed with de-ionized (DI) water, 30% HCl solution and again with DI water until the pH becomes 7.5 ml of di-ethyl ether was added to the material that remained after extensive washing and finally it was dried overnight at 60 °C.

### Synthesis of silica beads

The preparation of silica spheres was carried out by the previously reported Stöber method with slight modifications^56^. In a typical synthesis, ethanol-containing 3.01 M distilled water and 0.7 M ammonium hydroxide was measured into a beaker that was kept for stirring at room temperature. Another ethanol solution containing 0.22 M tetra-ethoxy orthosilicate was added dropwise to the previous solution. After further reaction for 6 h, the obtained microspheres were purified by centrifugation and washed with ethanol several times. Finally, the silica spheres were dried at 70° C for 5 h. The synthesized silica spheres have an average diameter of 385 nm (from the SEM image, Supporting Information Fig. S1).

### Optically assisted thermophoretic assembly

The experiments on thermophoresis were done on an optical trapping set up reported earlier^43^. In brief, an upright fluorescence microscope (Nikon Eclipse Ni-U with photoactivation unit) was utilized as the trapping platform. A 785 nm multimode diode laser (maximum power of 500 mW) was focused onto the sample through a 100 × oil immersion objective after collimation (Supporting Information Fig. S2). The power of the laser was limited to 100 mW at the entry of the objective. The samples were placed on the sample holder of the microscope and viewed through a CMOS camera (Nikon Digital Sight Fi3).

### Temperature mapping of laser illuminated GO microplatelets

In order to map the temperature, we used a quantitative phase microscopy based on a wavefront sensing technique named quadriwave lateral shearing interferometry, or more simply cross-grating (CG) phase microscopy. This technique uses a diffraction cross-grating positioned ~ 0.8 mm in front of a sCMOS camera (Zyla 5.5, Andor, Sid4-sC8, Phasics SA)^57^. The 3-dimensional temperature increase creates a refractive index gradient that distorts an incoming light wavefront (LED at 625 nm). This wavefront distortion is imaged through a 100 × oil immersion objective using CG microscopy, and then postprocessed using a deconvolution algorithm to retrieve the temperature distribution, with the only pre-knowledge of the temperature dependence of the refractive index of the liquid^58^. GO was drop casted on glass slides and heated using a Ti:Sapph laser set at 785 nm in continuous wave mode, focused at the entrance pupil of the objective to get a wide illumination of a GO structure.

### Bacteria culture and cytotoxicity/growth studies

#### Bacteria culture and preparation of graphene oxide sheet solution

The pure culture of *E. coli* was prepared by inoculating a single colony of *E. coli* from the master Luria Bertani (LB) agar (Himedia) plate to a conical flask containing 50 mL LB broth. After inoculation, the broth was kept at 37 °C overnight in shaker at 100 rpm. 500 µL of overnight grown *E. coli* culture was taken in a 1.5 mL microcentrifuge tube and centrifuged at 5000 rpm for 3 min. After centrifugation the supernatant was discarded and the pellet was washed with 1 × PBS twice. The pellet was diluted 4 times during experiment in order to maintain bacterial concentration. 1 mg/mL stock solution of GO sheet was prepared by dissolving GO sheets in Milli Q water and sonication for 20 min. After sonication, the stock solution was kept in a UV chamber for 20 min for sterilization.

#### Photothermal anti-bacterial property of GO

To investigate the anti-bacterial property of GO sheets, two different sets of bacterial suspensions were taken and mixed with various concentrations of GO sheets (20 µg/mL and 50 µg/mL) and put under the continuous wavelength laser of 785 nm having maximum power of 500 mW for 10 min and 20 min respectively in glass test tubes. After laser irradiation, the treated samples together with control were diluted 106-fold and 100 µL of each sample was plated on LB agar plate and kept at 37 °C for overnight in a biological oxygen demand (BOD) incubator for colony count assay.

#### Live/Dead Screening

10 µL of *E. coli* culture was taken on a glass slide containing GO layer and a microscope coverslip was placed on the slide with the help of a double-sided scotch tape to create a fluidic chamber of approximate thickness 100 µm. The glass slide was then placed on the microscope stage and the GO sheet was focused using 100 × oil immersion objective lens and illuminated with the 785 nm laser. The incident power of the laser at the output of the objective was 80 mW. The trapping events of bacteria were observed on the GO microplatelet using a CMOS camera (Nikon Digital Sight Fi3) In order to check the photoablation effect, 10 µL of propidium iodide (PI) having concentration of 8 µM was applied through one of the open ends of the coverslip and incubated for 10 min while maintaining the laser illumination on the GO. After 10 min of incubation with PI, washing was done using 1 × PBS and soaking the liquid from the other end of the coverslip with the help of tissue paper in order to eliminate any unbound PI. Images were then taken by using fluorescent microscope with laser irradiation at 532 nm and no irradiation as control.

#### Scanning Electron Microscopy (SEM) analysis

200 µL of each sample (control, 50 µg/mL of light exposed to the tube and 50 µg/mL without light exposure) were taken in a 1.5 mL micro-centrifuge tube and centrifuged at 9000 rpm for 10 min. The supernatant was discarded and the pellet was washed with 1 × PBS, followed by a centrifugation at 9000 rpm for 10 min. The supernatant was discarded. The pellet was then resuspended in 1 × PBS and 10 µL of sample was put on the slide for air drying. The slides were then covered with 2.5% glutaraldehyde and incubated at 4 °C for 2 h. The slides were washed three times with 1 × PBS followed by a series of alcohol wash with 30%, 50%, 75%, 90% and 100% ethanol for sample dehydration. The slides were air dried and taken for SEM analysis where the samples were sputter coated with a thin film of Pt/Au before imaging.

## Results and discussion

### Optically assisted thermophoretic assembly of silica beads

**Figure 1:**
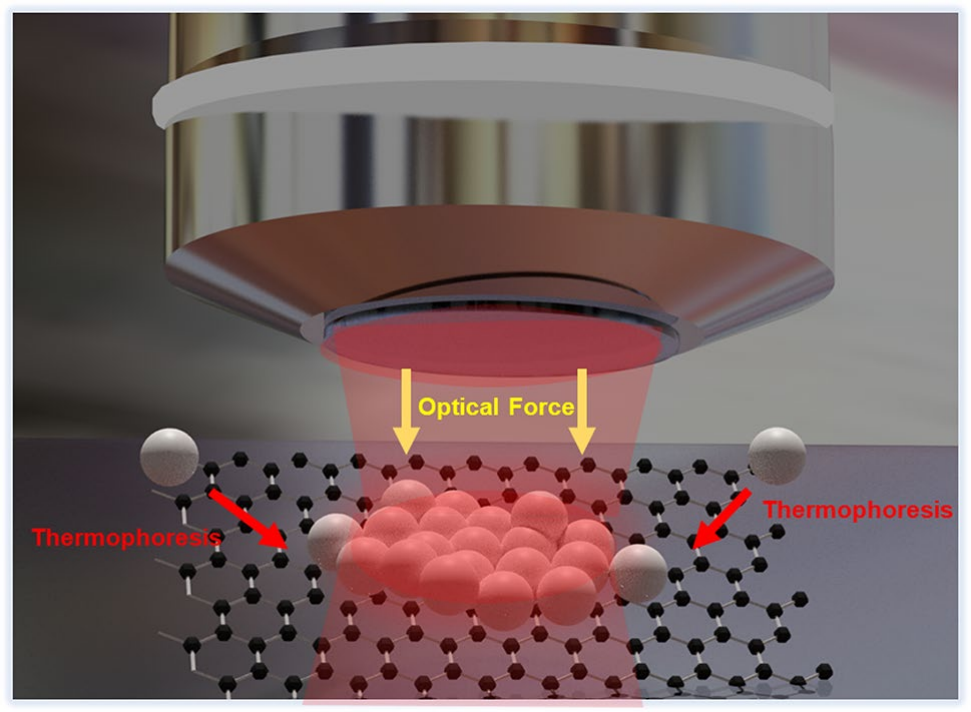
Schematic of the thermophoretic assembly of silica beads on GO. A 785 nm lser is focussed on a GO microstructure. The silica beads in aquous solution are attracted to the GO by long-range thermal forces generated by the temperature gradient due to photothermal effect. The beads get trapped on the surface and starts assemblying in an ordered fashion. Once the illumination is switched off, the particles are released back into the solution.

Thermophoretic experiments were performed as per the scheme shown in Fig. 1. GO was drop-casted on a pre-cleaned glass slide after sonication for 20 min. The glass slide was dried on a hot plate to obtain GO microplatelet samples of different sizes and thicknesses. A fluidic chamber was made using double-sided scotch tape of thickness 100 µm into which 10 µL of colloidal solution containing the silica beads was added. The chamber was sealed using a microscope coverslip (Supporting Information Fig. S2). The sample was placed on the holder of the optical trapping microscope and illuminated with a 785 nm multi-mode laser using a 100 × oil immersion objective (NA 1.3). The trapping events were visualized using a CMOS camera attached to the trapping microscope.

**Figure 2:**
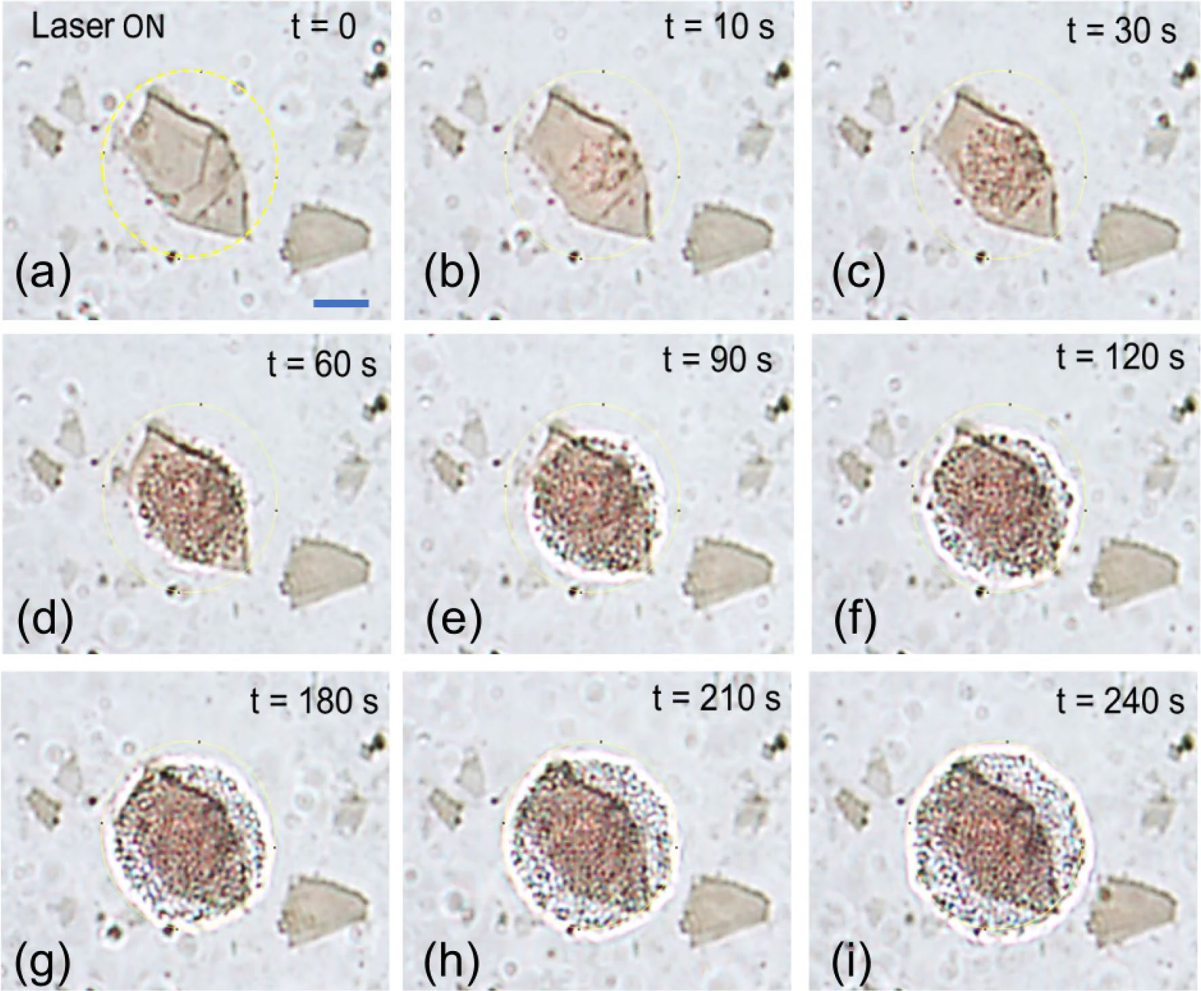
Video snapshots of optically-assisted thermophoretic assembly. Bright field video snapshots at different times of the experiment are shown. The yellow circle denotes the approximate illumination area. At *t* = 0, the laser illumination is switched on. At this moment, the silica beads are floating around the vicinity of the GO microplatelet. After few seconds, the beads start aggregating at the center of the GO. With time, there is an increase in the number of aggregated particles. After 240 s the growth has almost saturated and there is no further lateral increase in the area of aggregation. However, the beads still get attracted and sit on top of each other forming layers. It may be noted that the assembly of particles is restricted to the GO that has been illuminated with laser and unspecific binding was not observed. The laser intensity was kept at 80 µW/µm^2^. The scale bar in the first image (blue line) denotes 2 µm.

**Figure 3:**
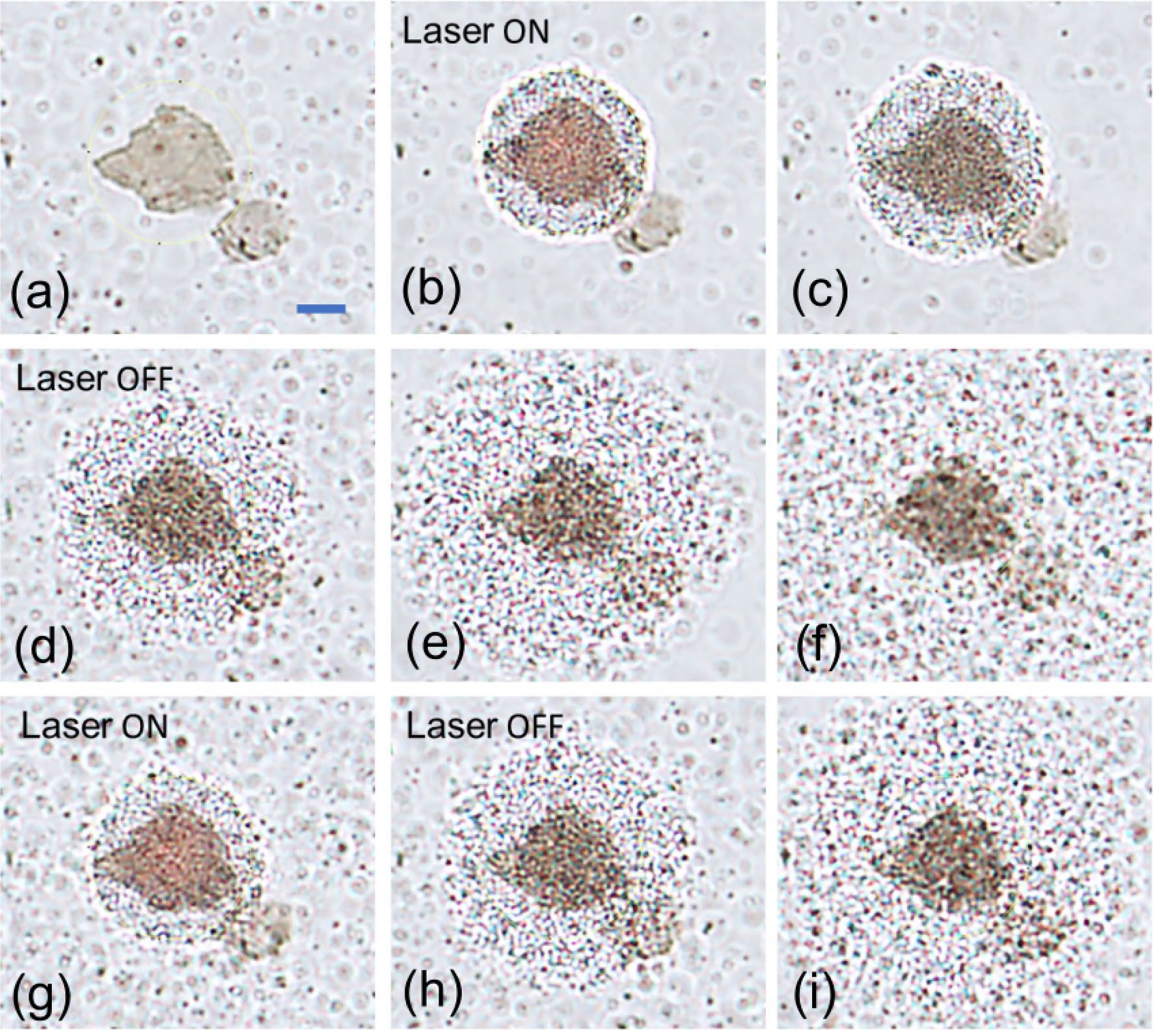
Reversible light assisted thermophoretic assembly of silica beads on GO. Bright field video snapshots at different times of the experiment are shown. The first image represents the time before switching on the laser. Within few tens of seconds of switching on the laser, a large assembly of silica beads are formed. As soon as the laser is switched off, the particles disperse back into the solution forming a swarm. Upon switching on, the particles immediately come back and aggregate on and around the illuminated GO. The process of assembly and redispersion into solution can be repeated several times simply by switching the laser on and off. The scale bar in the first image (blue line) denotes 2 µm.

In Fig. 2, video snapshots of the trapping and assembly of 385 nm silica beads is shown. The video is available in Supporting Information. Colloidal silica beads move around in the solution due to Brownian motion and are not trapped on GO sheet without laser illumination. When the laser light is turned on, silica beads in the vicinity of the GO platelet get trapped on the GO surface. With progress of time, a greater number of beads flow towards the GO and gets thermophoretically attracted to the hotter central section of the GO (the center). The assembly then grows in size as more and more particles get attached to the site. Apart from the thermophoretic attractive force, there is a convective flow of the fluid in vicinity of the illuminated GO microplate which brings more particles towards the assembly site. Within a time of 2 min, the assembly grows into a circular pattern with a diameter that is approximately equal to the illumination spot. The laser intensity was fixed to 80 µW/µm^2^ for the experiment. The assembly continued to grow with time and the size of the assembly was greater than the illuminated area. Apart from the lateral growth of the assembly in the sample plane, the beads also got trapped on top of the first layer forming multilayers of beads. It was observed that the silica beads that were at the center of the assembly (region of the illuminated GO microplate) were strongly fixed to their position compared to the outer layer of beads. The particles on the outer part of the assembly showed larger Brownian motion (See Supporting Information Movie1). On switching off the laser illumination, the particles were immediately released back into the solution forming a large swarm of particles. Once the laser is switched on, the particles assemble back on the substrate rapidly as the population of particles around the trapping site is large. Again, on switching off, the particles are released back into the solution (Supporting Information Movie2). The video snapshots of this trapping and releasing events is shown in Fig. 3. These observations indicate that the silica beads are not attached to the substrate by any weak forces such as van der Waals but trapped with opto-thermal forces. Reversible assembly and releasing were repeated several times by switching the laser on and off respectively. The experiments were performed on glass substrates without GO in order to confirm that the large-scale assembly process was indeed due to the illuminated GO. Although some silica beads were trapped on the glass substrate due to optical forces, we did not observe any large-scale assembly of the beads.

**Figure 4:**
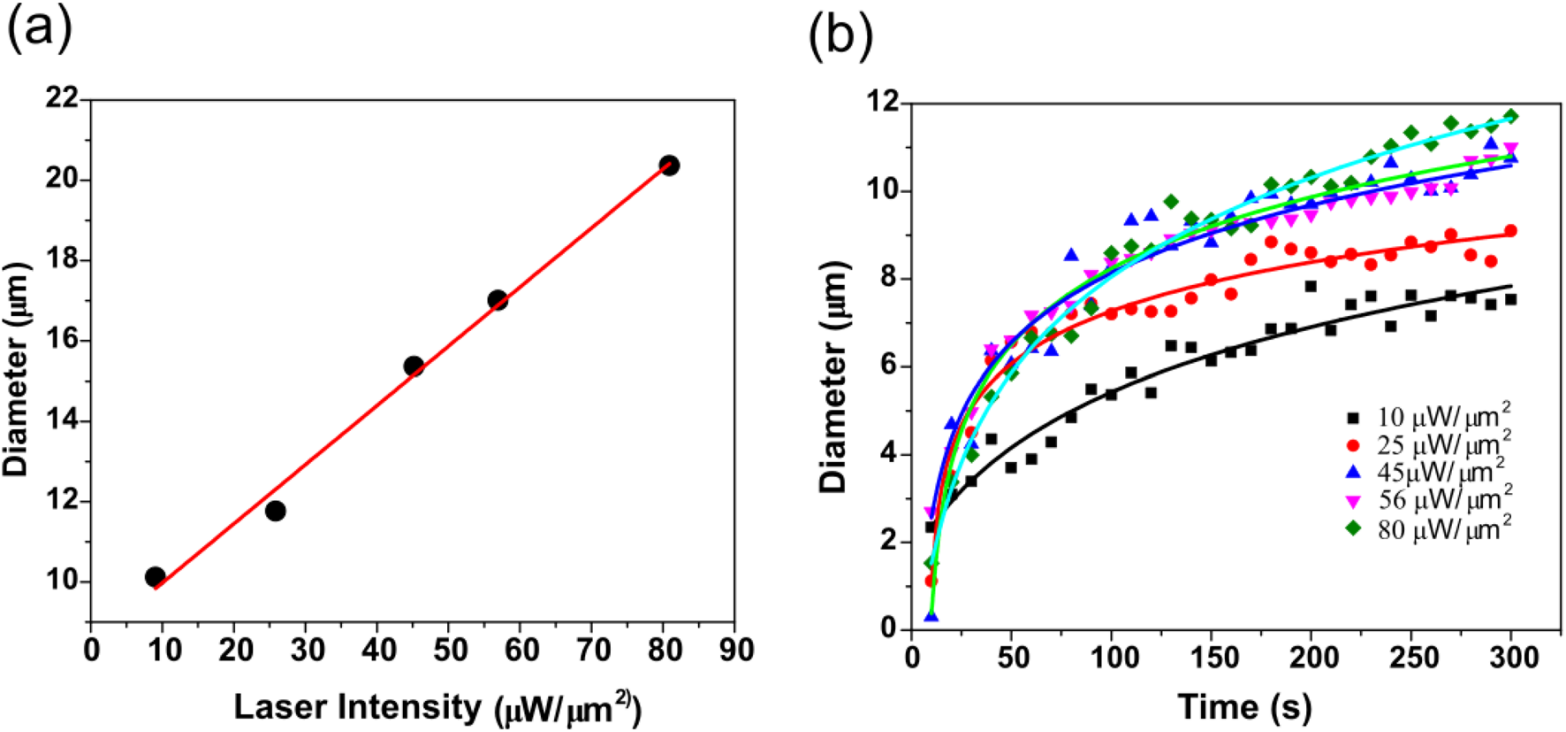
Dependence of the size of particle assembly on the laser intensity and time. The size measurements were taken after a time of 5 min (**a**) As the laser intensity is increased the diameter of the aggregation increases in a linear fashion. Beyond 80 µW/µm^2^, there was strong convection currents and the assembly of nanoparticles became unstable. Moreover, the GO microplatelet was damaged at high laser intensities. (**b**) Time dependency on the size for various laser intensities is plotted. On switching on the laser, there is a rapid assembly of the silica beads which kept increasing with time. However, the rate of assembly process soon saturated after which there was little change in the size. A logarithmic fit to the points is shown in the plots.

A plot of the laser power (intensity) versus the size of assembly (diameter of the circular region) of the particles is shown in Fig. 4a. Here, the laser intensity was gradually increased after every 5 min and the assembly size was noted. It can be observed that as the laser power is increased, the area of assembly of the beads increases in a linear fashion. This indicates that the absorption of light by graphene oxide is indeed linear with the power of the light yielding a monotonous increase in the local temperature due to photothermal effect. Other factors such as thickness of the GO microplatelet, availability of silica beads in proximity of the GO and thermal conductivity of the solvent also play a significant role. We limited the laser intensity to less than 100 µW/µm^2^ as it was observed that increasing the intensity further damages the GO platelet. Furthermore, laser intensities larger than 100 µW/µm^2^ lead to higher convection and destabilization of the particle assembly. In all our experiments the GO with sheet thickness was limited to 20 nm and de-ionized water was used as solvent. The growth of the assembly is very rapid at the beginning once the illumination is turn on and then saturates with time. This can be easily noted from Fig. 4b where the diameter of the assembly is plotted against the time for fixed laser powers. A logarithmic growth function is fitted to the experimental points in the plot. The growth pattern is similar for all the incident laser powers but larger powers yielded larger assembly size.

It has been reported earlier that noble metal nanostructures can aid in thermophoretic assembly by means of the excitation of surface plasmon and joule heating^59^. There have been several reports of harnessing the plasmonically-generated thermal forces for trapping and assembly of nano as well as micro particles. Recently a large-scale assembly of silica microbeads using gold platelets have been demonstrated^26,39^. In our case, the photothermal effect of GO structure is responsible for the local heat generation and the colloidal assembly process. Apart from thermophoretic force, convective and thermo-osmotic forces can play a significant role in such extended assembly of colloids in the vicinity of a heat source. In order to confirm that the actual mechanism of assembly is optically-assisted thermophoresis and not pure convection effect, we performed the trapping experiments with a very small chamber thickness so as to reduce the convection currents^26^. By placing the top coverslip directly on the solution (without the spacer tape), it was possible to obtain chamber thicknesses of only a few micrometers after adding 10 µL of the colloidal solution. Even at such low chamber depths, where convection currents are minimal due to restricted vertical temperature gradient, trapping and assembly of silica beads were observable similar to the previous experiments. The experiments were also done with polystyrene beads (PS) of size 500 nm instead of silica beads. In the case of PS (Sigma Aldrich, carboxylate-modified) it was observed that at low powers few PS beads which were in close vicinity of GO microplatelet were attracted towards the GO by optical forces but large-scale assembly could not be achieved. By increasing the incident laser power, it was observed that the PS beads moved away from the GO due to convective forces unlike the case of silica beads. It can be implied from these observations that the origin of force for large scale assembly process is thermophoretic in nature rather than convective. Thermo-osmotic forces can also be neglected in this process as we did not see the aggregation of PS beads on the heated GO^60^. Thus, we believe that thermophoresis plays an important role in such large-scale processes.

In order to compare the efficiency of photothermal capability of GO with plasmonic metals such as gold, we performed similar opto-thermophoretic experiments using gold microplatelets as reported by Sharma et al.^39^. Unlike their work where the gold structure was illuminated under Kretchmann geometry, we used direct illumination of the gold structure to generate the temperature gradient. Large gold microplatelets were synthesized using chemical reduction methods reported earlier^61^. The gold structures were drop casted on precleaned glass slides followed by the addition of the solution containing silica beads. As in the case of GO microplatelets, the triangular gold platelet structure was illuminated by the 785 nm laser using a 100 × oil-immersion objective and the assembly process was observed for comparable durations. The power and spot size (10 µm approximately) of the laser beam was fixed to the similar parameters used in the GO experiments. It was observed that although there was trapping and assembly of the silica beads on the gold structure, the process was much slower compared to the GO aided assembly for the same laser intensity. Furthermore, at the incident power used in the experiment, we observed that the assembly was limited to the dimension of the gold microstructure (Supplementary Information Fig. S3). When gold nanostructures with GO sheets on top of them were illuminated, it was seen that there was rapid assembly of silica beads. This is expected as there is increased absorption due to the presence of GO on the gold structure. These experiments lead to the observation that GO can be a good substitute to plasmonically-generated thermal gradients. Apart from the ease of synthesis, one major advantage of using GO for such optically-induced thermophoretic applications over gold substrates is that it is possible to observe the sample in transmission, because GO is semitransparent while gold flakes are opaque. This is particularly useful when fluorescence study of the trapped particle is of interest.

### Temperature mapping of laser illuminated GO structures

Thermophoresis is related to the spatial temperature gradient rather than the temperature difference or absolute temperature at any point. To understand the photothermal properties of GO, we performed temperature measurements using CG microscopy^62^. This technique has been previously used to study the photothermal properties of metallic nanowires and other nano-objects^63,64^. The brief description of the method is given in the experimental section. The change in the refractive index due to local temperature increase is converted to a wavelength distortion and then postprocessed using a deconvolution algorithm to retrieve the temperature distribution. The algorithm is based on a Green’s function formalism. Let us define $G_{\ell } \left( {x,y} \right)$ the wavefront profile distortion created to a point-like source of heat. Its expression is a simple logarithmic function. Then, the wavefront profile created by an arbitrary 2-dimensional heat source density $p\left( {x,y} \right)$ can be expressed as a convolution with $G_{\ell }$: $W\left( {x,y} \right) = \left[ {G_{l} \otimes p} \right]\left( {x,y} \right)$. By inverting this equation using the measured W distribution, one ends up with the distribution of heat source density $p$. Finally, p is convoluted with the thermal Green’s function of the system defined by $G_{T} \left( {x,y} \right) = 1/\left( {4\pi \kappa \sqrt {x^{2} + y^{2} } } \right)$, i.e., $T\left( {x,y} \right) = \left[ {G_{T} \otimes p} \right]\left( {x,y} \right)$.

**Figure 5:**
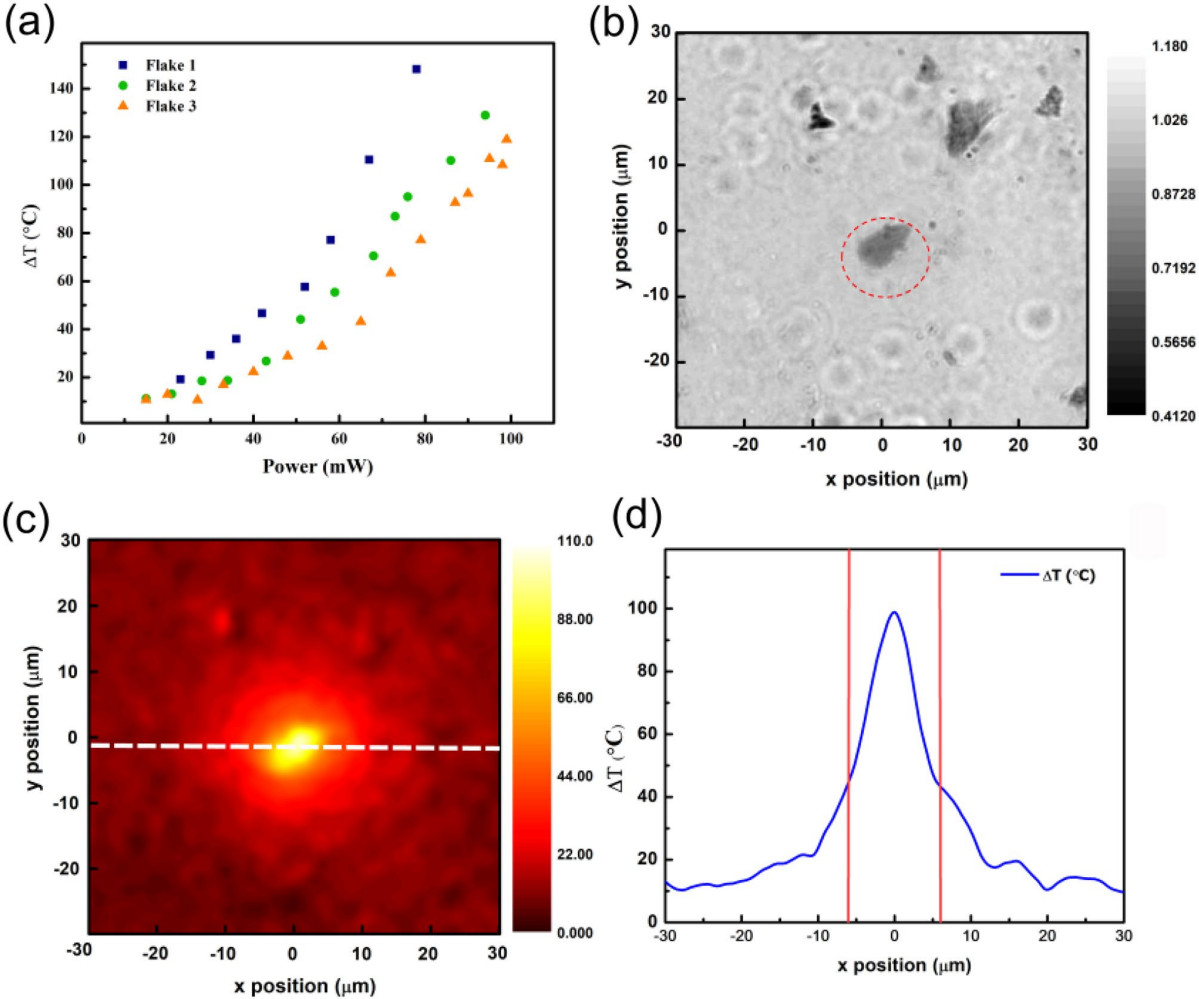
Temperature mapping of illuminated GO using CG phase microscopy. (**a**) A plot of the change in temperature $\Delta T$ versus the incident laser intensity is shown for three different GO flakes. (**b**) Transmission map of a typical GO microplatelet as obtained from the set up. (**c**) Temperature map of the GO. A temperature change of more than 100 °C was observed for an illumination of 60 mW power (**d**) The temperature change profile of (**c**) showing the actual change in temperature along the *x* direction.

In Fig. 5a, the dependence on the increase in temperature with the incident laser power of three different GO microplatelets are plotted. Interestingly, it can be observed that the increase in temperature is linear for initial lower temperatures while it becomes steeper at higher incident laser intensities. It has been reported that exposure to laser light can lead to the reduction of GO thereby altering its optical properties^65^. GO has predominantly *sp*^3^ bonded C-O domains and relatively smaller *sp*^2^ bonded atoms while reduced GO tends to be more graphene-like in its properties. This change is evident in the transmission measurements as well. After a threshold power, the optical properties of the GO tend to change as observed in Fig. 5a. The temperature rise is also dependent on the thickness of the GO and the properties of the medium (water). It can be observed that the local temperature can easily reach above 100° C for incident optical powers as low as 60 mW. It is worth noting that even at this temperature, we do not observe boiling. This can be attributed to the superheating effect that was observed in the case of plasmonic heating by gold nanostructures^66,67^. A transmission map image of the GO platelet is shown in Fig. 5b. The corresponding temperature map for the structure is given in Fig. 5c. A strong temperature gradient can be noticed in the vicinity of the GO structure. A profile taken along the x-axis of the temperature map (Fig. 5d) shows that it is possible to obtain high local temperature gradient by illuminating the GO with a laser light.

### Opto-thermal assembly of *E. coli* bacteria and photothermal ablation studies

Photothermal properties of graphene and its derivatives have been exploited in applications such as photothermal cancer therapy and photoinactivation of bacterial specimens. However, there have been no reports on large scale thermophoretic assembly of bacteria and their photothermal destruction using GO. To extend the application of our scheme of thermophoretic assembly using GO to biological samples, we chose to work with *E. coli* bacteria.

**Figure 6:**
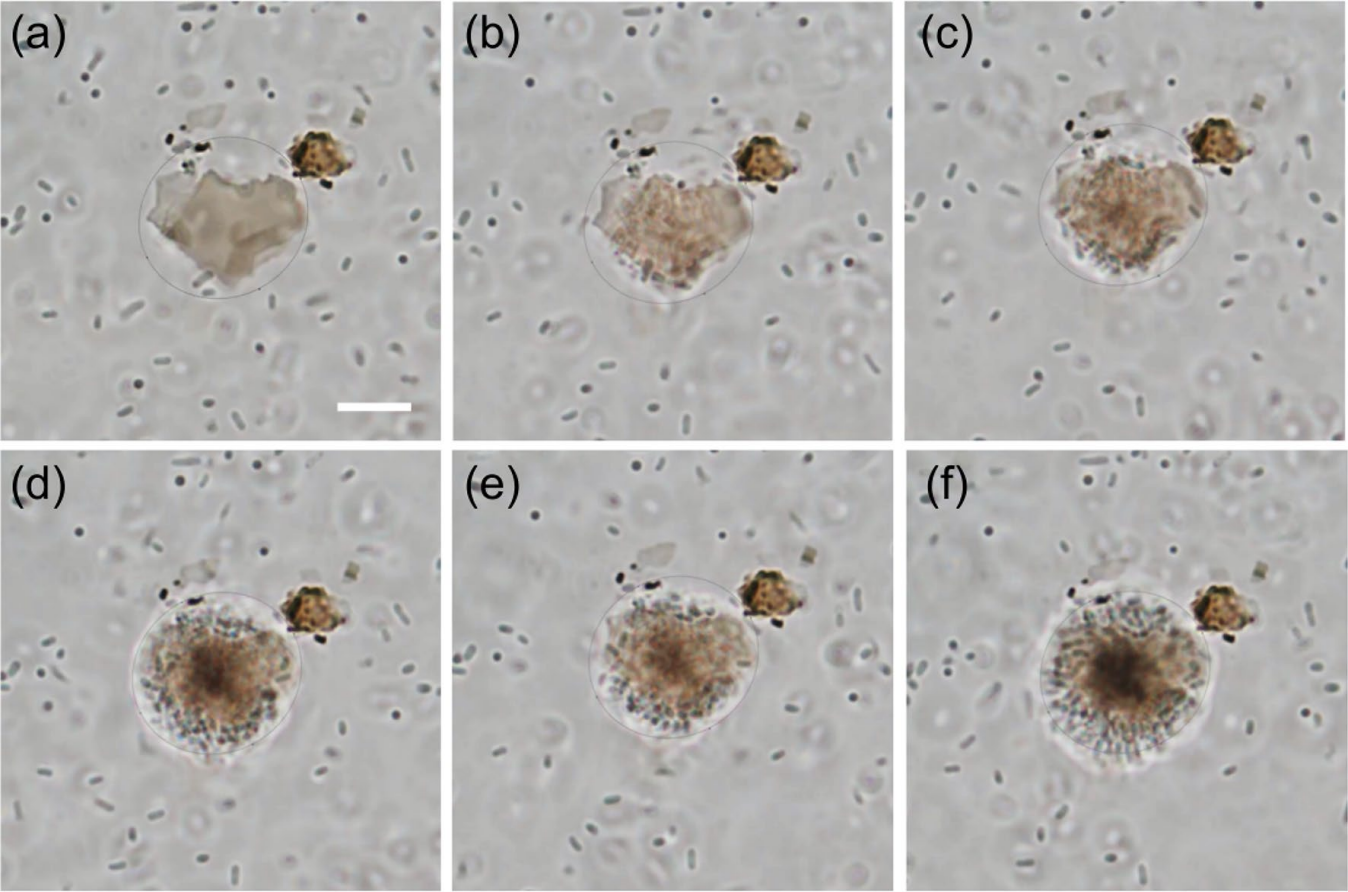
Thermophoretic assembly of *E.coli* bacteria on GO (**a**) Brightfield microscopic video snapshots of trapping and assembly of *E.coli* bacteria before the laser was switched on. A suspension of bacterial solution can be seen in the image. (**b**)-(**f**) video snapshots after switching on the laser. The bacteria come in the vicinity of the illuminated GO and gets trapped. The assembly grows larger with time. A large circle of bacterial assembly is formed after few seconds of illumination. The laser intensity was kept at 50 µW/µm^2^ for the experiment. The scale bar in the first image (white line) denotes 2 µm.

**Figure 7:**
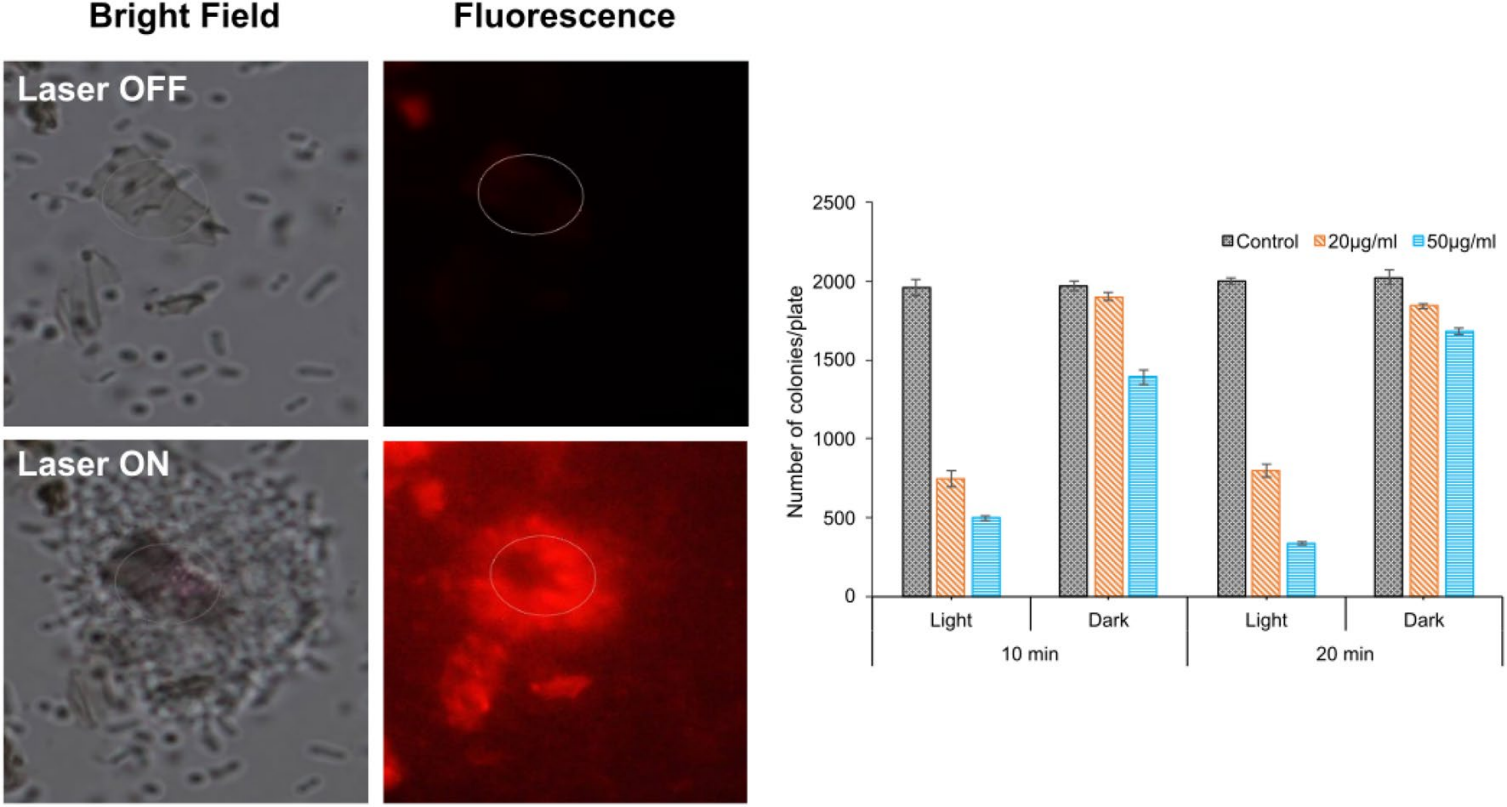
Growth and Cytotoxicity assay of *E. coli*. (**a**) Bright field image of GO with the bacterial solution. The bacteria can be seen moving around randomly in the solution. (**b**) Fluorescence image of (**a**) with PI added to the solution. Dark image implies there are no dead bacteria before laser illumination. (**c**) Brightfield image of the sample after few minutes of laser irradiation. Bacteria can be seen trapped on the GO. The laser intensity used in the experiment was 50 µW/µm^2^. (**d**) Fluorescence image of (**c**) shows that the trapped bacteria are dead. PI staining allows to observe dead bacteria. Due to the large temperature gradient near the GO, the bacteria were photo-ablated. (**e**) Viability of *E. coli* on LB agar plates after exposing them to laser irradiation (80 µW/µm^2^) for 10 and 20 min with different concentrations (20 µg/mL and 50 µg/mL) of GO sheets.

In Fig. 6, a sequence of video snapshots of the trapping and assembly of *E. coli* bacteria using GO are shown. As in the previous experiments, the laser intensity was fixed to 80 µW/µm^2^. It can be clearly observed that with time a greater number of bacteria come towards the illuminated GO site and forms an assembly. In order to check the photothermal bactericidal efficiency of GO, live-dead cell viability test was performed. Figure 7a shows the trapped bacteria on the GO microplatelet that was illuminated with the laser. After PI staining, only the dead bacteria are visible in the fluorescence image as it stains on the dead bacteria. It can be observed that the bacteria that got aggregated on the laser illuminated GO were photo-ablated.

In order to verify the photoablation and bactericidal effect of laser illuminated GO, a solution of bacteria mixed with GO in a tube was irradiated directly with 785 nm laser at 500 mW for 10 and 20 min. A control sample was also prepared together but without laser illumination. The above treated bacteria were then cultured on plates for growth count studies.

Inhibitory effect was confirmed on cells on LB agar plate treated with 20 µg/ml and 50 µg/ml concentrations of GO sheets (Fig. 7) with light irradiation (80 µW/µm^2^). Cells without irradiation show no inhibition and the number of colonies obtained in agar plate at both concentrations correspond to the control sample. However, when the cells were treated with 10 min light irradiation, 20 µg/ml and 50 µg/ml GO sheets, the colony count was 55% decreased (Fig. 7). The antibacterial effect was more obvious in 20 min light irradiated samples of GO sheets at both 20 µg/ml and 50 µg/ml concentrations and the colony count was further decreased 75% and 85%, respectively (Fig. 7). These results clearly indicate time and dose dependent phototoxic effect of GO sheets. These facts were further confirmed by a bacterial growth study that was done on the irradiated samples (Figures S4 and S5). It was observed that the colony growth of *E. coli* on the irradiated sample was much lower than that of non-irradiated samples. Furthermore, higher concentration of GO or longer exposure to the 785 nm laser resulted in a stronger photoablation and hence a smaller colony growth.

**Figure 8:**
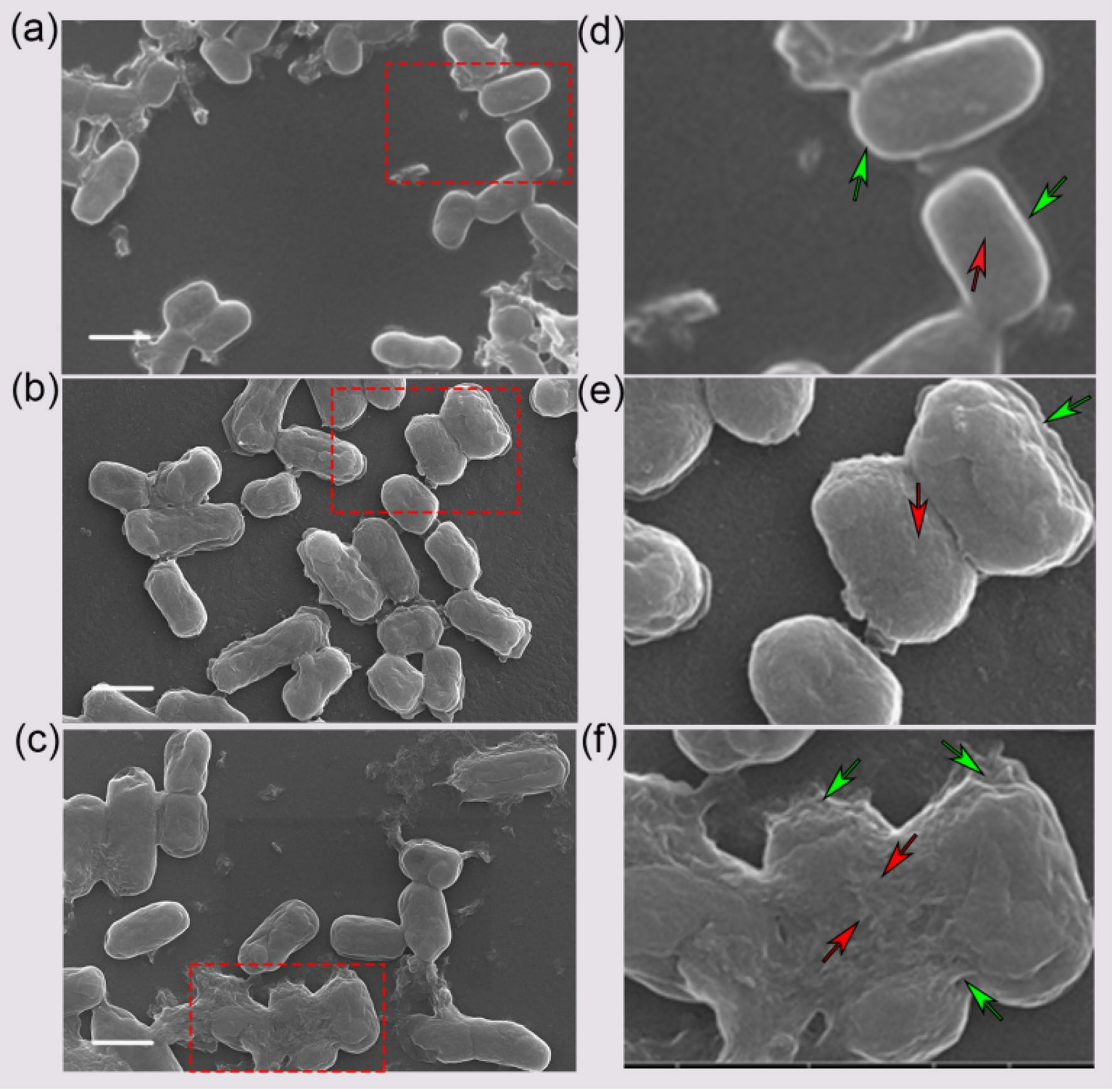
SEM images of photo ablated *E. coli*. (**a**) *E.coli* without GO sheets and light irradiation. (**b**) *E.coli* treated with GO sheets but without laser irradiation. (**c**) *E.coli* treated with GO sheets irradiated with laser (785 nm, 80 µW/µm^2^) for 20 min. Panel (**d**–**f**) represents the zoomed images corresponding to the red circle in the image (**a**–**c**). The red arrows indicate the areas on the membrane and green indicates the outer membranes. Scale bar, 2 µm.

From the SEM images, it was observed that morphological changes and cell damage in the cells in photoactivated GO treated *E. coli* cells, was due to the photothermal effect of GO sheets (Fig. 8a–f). On the other hand, very mild morphological changes were observed in the control cells and the cells treated with GO sheets without photoactivation. Cell damage in Fig. 8c–f is obvious and the cells are clearly showing damage in the zoomed panel indicated with red arrows. These data clearly indicate the photothermal effect of GO sheets against *E. coli* cells.

## Conclusions

In summary, we demonstrated the capability of GO 2D microstructures for large assembly and swarming of silica beads. Photothermal studies on GO microplatelets showed that it is possible to use GO structures as micro-heat sources for thermophoresis. It was possible to reversibly trap and assemble silica beads of different sizes on and around the GO site. There is a linear increase in the size of the assembly with the laser power. The initial growth of assembly is rapid and then saturates with time. It was also observed that the particles at center of the trapping site we tightly fixed while those on the periphery of the assembly were dangling due to large Brownian motion. Temperature mapping studies on GO microplateles revealed the excellent photothermal capabilities of these 2D materials. An increase in temperature of 100 °C in the vicinity of the illuminated GO could be easily achieved at laser intensities of (80–100 µW/µm^2^). Ease of large-scale synthesis and favorable optical properties of GO makes this scheme a good replacement for plasmonic based heating systems. In order to demonstrate the biological application of the GO based thermophoretic assembly scheme, we trapped *E. coli* bacteria and performed studies on photothermal effects on the trapped bacteria. This work presents a promising scheme for using graphene based 2D materials as heat sources for thermophoretic assembly and photothermal applications. It is envisaged that this scheme will prove useful in assembling plasmonic nanoparticles for spectroscopic applications such as surface-enhanced Raman scattering and fluorescence studies.

## Supplementary Information

Supplementary Information 1.

Supplementary Video 1.

Supplementary Video 2.
