# Supplementary material for: Optically-assisted thermophoretic reversible assembly of colloidal particles and E. coli using graphene oxide microstructures
Source: Sci Rep. 2022 Mar 7;12:3657. doi: 10.1038/s41598-022-07588-4 (PMC8901786; doi:10.1038/s41598-022-07588-4)
Supplement: Supplementary file 1 — Supplementary Information 1. [file 41598_2022_7588_MOESM1_ESM.docx]

Supporting Information

Optically-assisted thermophoretic reversible assembly of colloidal particles and *E.coli* using graphene oxide microstructures

*Jostine Puthenveetil Joby^1^, Suman Das^2^, Praveenkumar Pinapati^1,^ Benoît Rogez^3^, Guillaume Baffou^3^,* *Dhermendra K. Tiwari ^2*^ and Sudhir Cherukulappurath^1*^*

^1^School of Physical and Applied Sciences, Goa University, Taleigao Plateau, Goa 403206, India

^2^Department of Biotechnology, Goa University, Taleigao Plateau, Goa 403206, India

^3^Institut Fresnel, CNRS, Aix Marseille University, Centrale Marseille, Marseille, France

*Address correspondence to: [sudhir.c@unigoa.ac.in](mailto:sudhir.c@unigoa.ac.in), dhermendratiwari@gmail.com


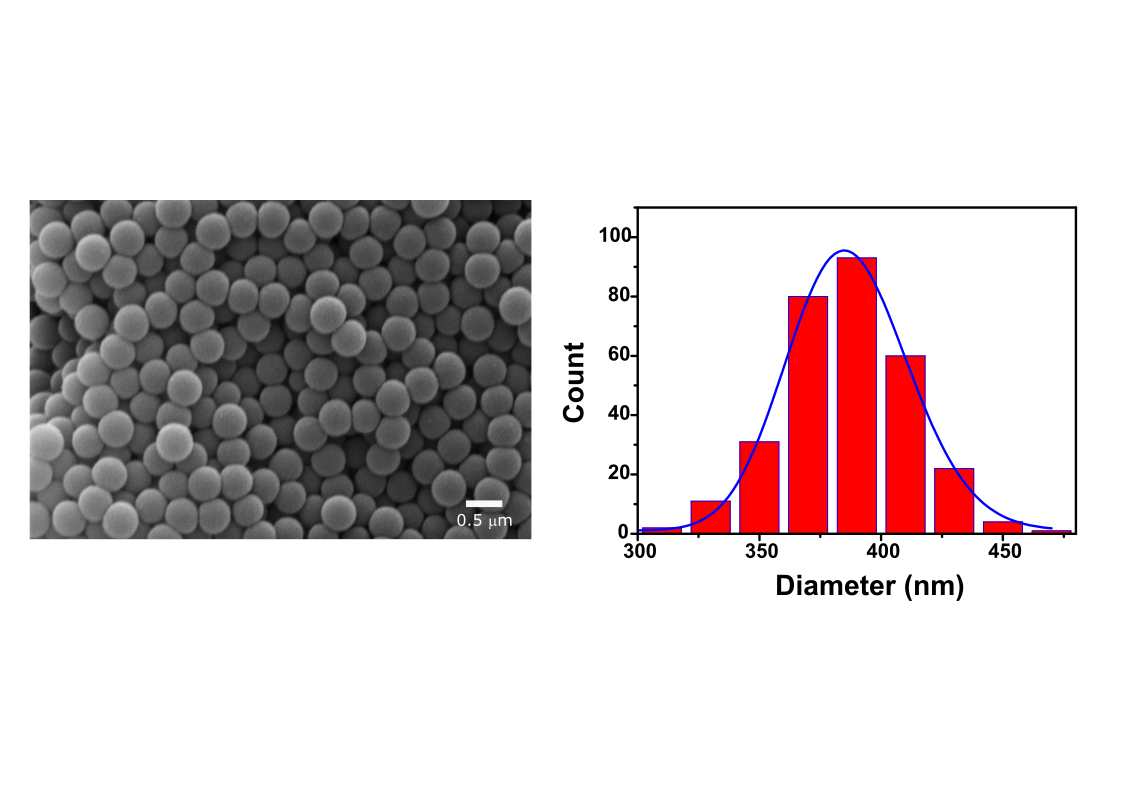


**Figure S1**. (a) SEM image of the chemically synthesized silica nanospheres (b) From the size distribution histogram, it can be observed that the silica nanospheres have an average diameter of 385 nm.


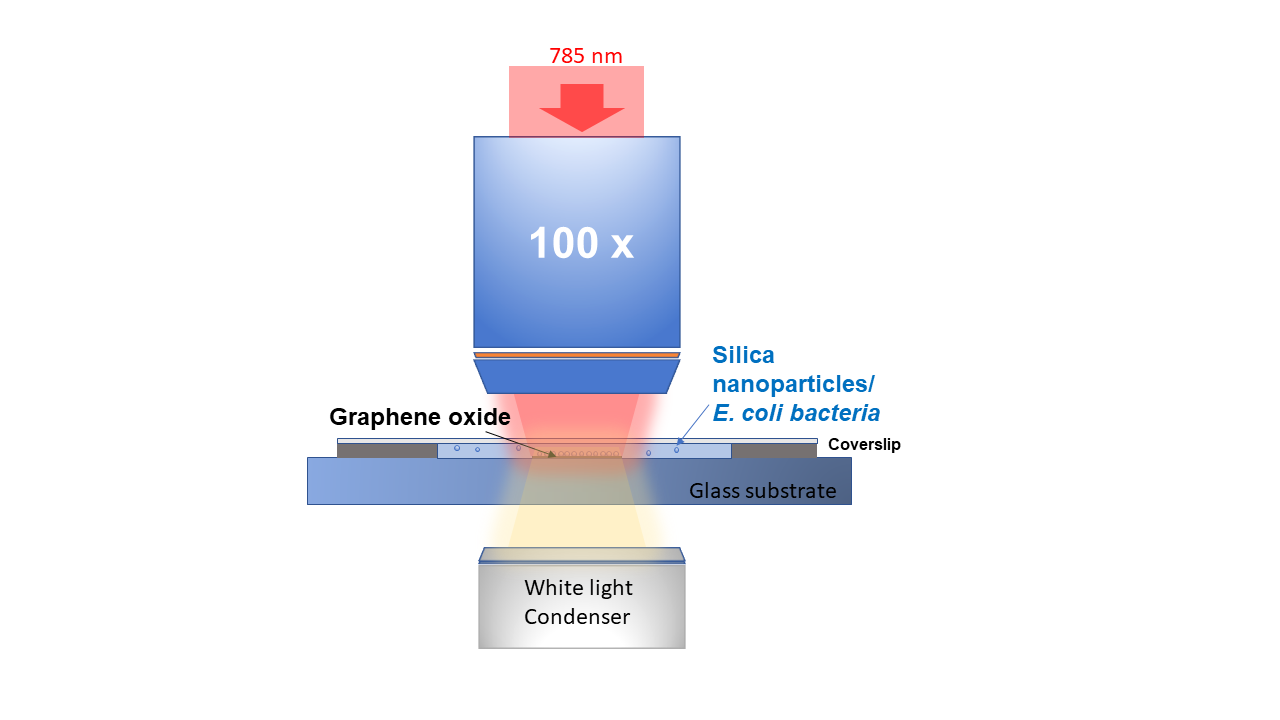


**Figure S2**. Schematic depicting the experimental set up for the GO-assisted thermophoresis. A drop of the sample containing silica beads or *E. coli* is placed on a glass slide with GO coated on it. A thin micro coverslip is then placed on top of the solution after putting double-sided tape on the glass slide to make a fluidic chamber of 100 μm. The glass slide sample is then mounted onto an upright microscope wherein it is illuminated with a 785 nm CW laser using a 100x oil objective. A white light illumination from below is used to view the GO microstructures and the thermophoresis event. A CMOS camera mounted on top of the microscope images the sample.


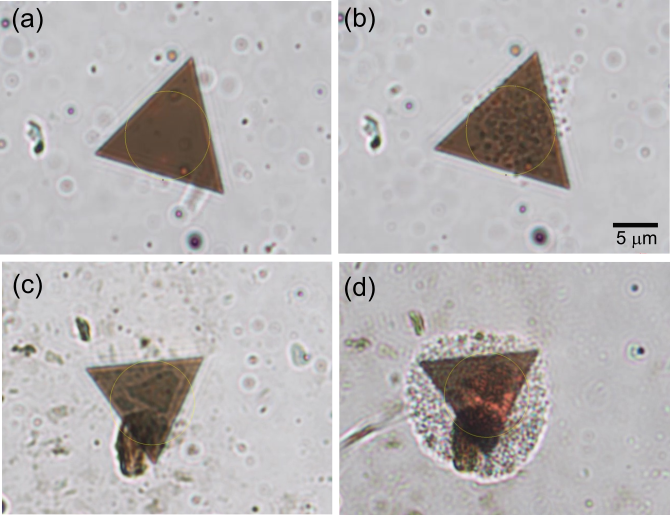


**Figure S3**. Thermophoretic assembly with gold microplatelets (a) A gold triangular microstructure that is illuminated with 785 nm laser. A drop of silica particles (average diameter of 385 nm) is added on the glass slide. (b) After 5 minutes of laser illumination silica nanoparticles assemble on the illuminated region of the gold structure. (c) A gold microplatelet with graphene oxide (GO) film on top. In this case, it can be seen that there is larger assembly of silica nanoparticles compared to (b). The experimental conditions such as laser intensity and spot size were kept similar in both cases.


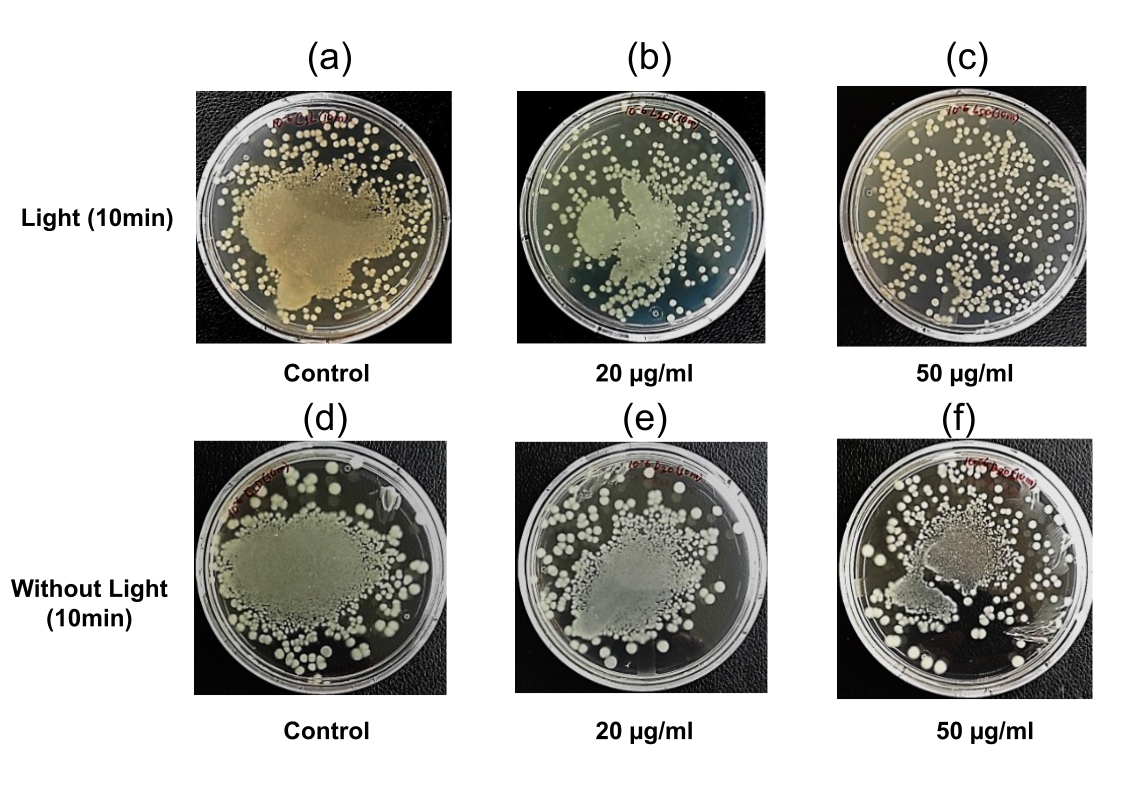


**Figure S4**. Colony images of *E.coli* that was incubated with GO and irradiated with 785 nm laser for 10 minutes. (a) Control sample with no GO but illuminated with laser. (b) and (c) represent the colony growth images for GO concentrations of 20 and 50 μg/ml. Figures (d)-(e) represent similar bacterial growth images but without any laser irradiation. It may be noted that GO by itself can be antibacterial at higher concentrations.


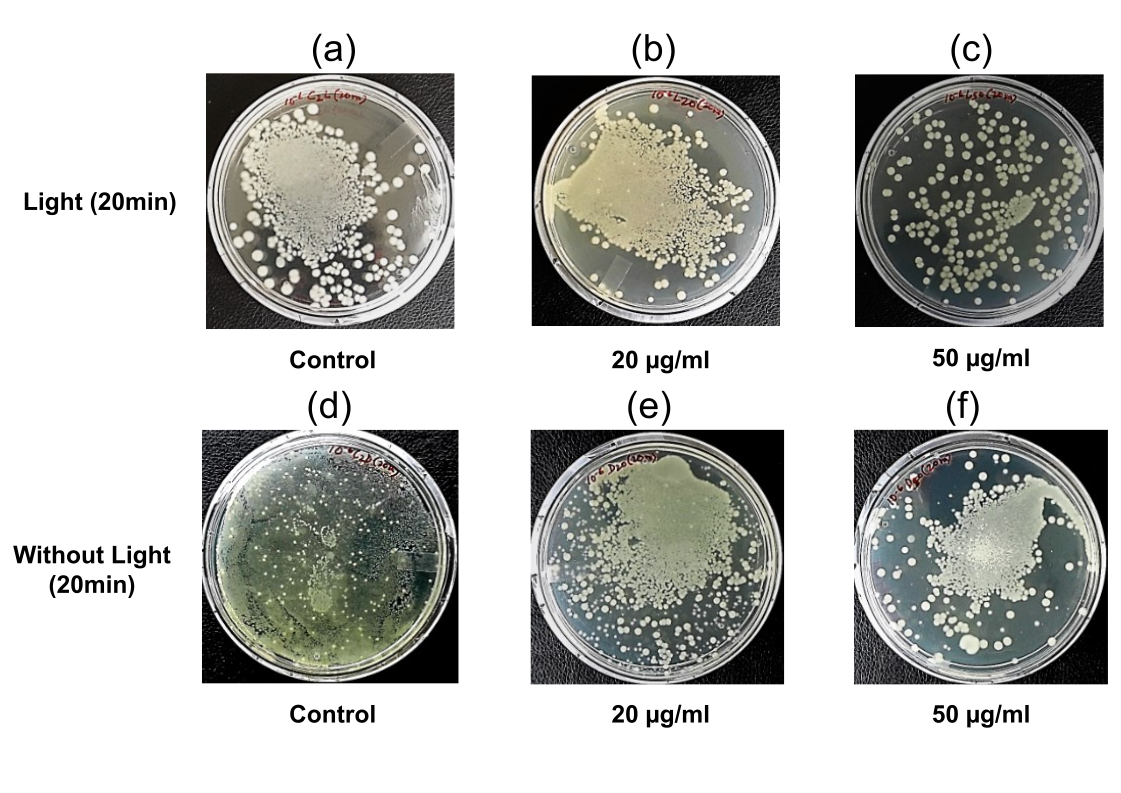


**Figure S5**. Colony images of *E.coli* that was incubated with GO and irradiated with 785 nm laser for 20 minutes. (a) Control sample with no GO but illuminated with laser. (b) and (c) represent the colony growth images for GO concentrations of 20 and 50 μg/ml. Figures (d)-(e) represent similar bacterial growth images but without any laser irradiation. It may be noted that GO by itself can be antibacterial at higher concentrations.

**Supplementary Movie 1 (file SI_V1):**

Experimental demonstration of optically-assisted thermophoretic assembly of silica beads on GO microstructure. The silica beads have an average size of 385 nm. A 785 nm multimode laser was used as illumination source to generate photothermal effect on the GO. The bead assembly starts from the GO and extends well beyond the structure. The laser intensity used in the experiment was 80 μW/μm^2^. The scale bar in the first image (blue line) denotes 2μm.

(The movie is shown in 10x speed for reducing file size.)

**Supplementary Movie 1 (file SI_V1):**

Experimental demonstration of reversible assembly of silica beads on GO microstructure using thermophoretic forces. The experimental conditions were similar to that of in Supplementary Movie 1. When the laser light is switched off, the beads are released back into the solution. On switching the laser on, the silica beads start re-assembling on the GO. This reversible assembly and release can be repeated several times. The laser intensity used in the experiment was 80 μW/μm^2^. The scale bar in the first image (blue line) denotes 2μm.

(The movie is shown in 10x speed for reducing file size.)
